# Diagnostic Accuracy of a Machine Learning Model for Cervical Vertebra-Based Skeletal Maturity Assessment in Pediatric and Adolescent Patients Using Cephalometric Radiographs

**DOI:** 10.3390/diagnostics16182886

**Published:** 2026-09-08

**Authors:** Narmin Helal, Faisal Al-Malki, Basil Saadi, Osama Basri

**Affiliations:** 1 Pediatric Dentistry Department, Faculty of Dentistry, King Abdulaziz University, Jeddah 21589, Saudi Arabia; 2 Faculty of Dentistry, King Abdulaziz University, Jeddah 21589, Saudi Arabia; faisalmalki2000d@gmail.com (F.A.-M.); basilsaadi13@outlook.com (B.S.); 3 King Faisal Specialist Hospital and Research Center, Jeddah 21499, Saudi Arabia; obasri@gmail.com

**Keywords:** cervical vertebral maturation, skeletal maturity, artificial intelligence, cephalometric radiography, orthodontics, deep learning

## Abstract

**Background/Objectives:** Skeletal maturity assessment is essential for timing orthodontic growth-modification treatment. The cervical vertebral maturation (CVM) method is widely used, but manual staging is subjective and prone to inter-observer variability, and artificial intelligence (AI) may improve its consistency and accuracy. The aim of this study was to develop and externally validate a fully automated deep-learning pipeline for CVM assessment and to determine its diagnostic accuracy for three-phase and six-stage classification against calibrated expert staging. **Methods:** A cross-sectional three-stage deep-learning pipeline was trained and internally cross-validated on 523 cephalometric radiographs from King Abdulaziz University. Images were CLAHE-enhanced; YOLOv8 detected the C2–C4 region; a ResNet-50 classifier assigned the growth phase (Early, Peak, Late); and phase-specific binary classifiers assigned CVM stages (CS1–CS6). External validation used the independent ‘Aariz dataset (*n* = 150). **Results:** The detector achieved mAP@0.5 = 0.9939 and mean IoU = 0.8884. On external validation, the three-phase classifier reached 96.0% accuracy, macro-F1 0.919, weighted κ 0.951 and ROC-AUC 0.998, whereas the end-to-end six-stage cascade reached 79.3% accuracy, macro-F1 0.735, weighted κ 0.910 and ROC-AUC 0.923. Most errors occurred between adjacent stages, with 98.7% of predictions within ±1 stage of the reference standard. **Conclusions:** The automated pipeline showed promising performance for three-phase CVM classification and moderate performance for six-stage classification on one independent external benchmark. Further multicenter prospective validation is required to establish its generalizability and clinical utility.

## 1. Introduction

Effective orthodontic treatment and planning include assessing skeletal maturity, as this helps clinicians identify the optimal timing for growth-modification interventions [1].

Accurately determining a patient’s developmental stage is crucial in the pediatric population, where intervention timing is critical for guiding craniofacial growth and achieving stable treatment outcomes, necessitating precise, objective assessment methods to inform clinical decisions [2].

Conventional methods such as chronological age and hand–wrist radiographs have been used, but they exhibit weak correlation with biological maturity and entail unnecessary radiation exposure, increasing patient risk without necessarily improving diagnostic precision [3].

The CVM method assesses skeletal maturation from the morphology of the C2–C4 vertebrae visible on lateral cephalometric radiographs routinely obtained for orthodontic diagnosis, thereby avoiding the need for an additional hand–wrist radiograph [1,4,5]. Artificial intelligence has subsequently been applied to automate CVM staging and reduce the variability associated with manual assessment [6,7].

Despite its clinical utility, manual CVM assessment has significant drawbacks.

Manual staging of cervical vertebral morphology can be time-consuming and prone to inter- and intra-observer variability, thereby affecting reproducibility and diagnostic accuracy [6,8]. These drawbacks highlight the need for more objective and standardized approaches to skeletal maturity assessment that yield consistent results across clinical settings.

In several studies, AI has become a valuable tool in medical imaging, enabling automated and standardized evaluation of morphological features, reducing observer bias, and improving diagnostic efficiency [9]. Deep learning models have demonstrated the ability to detect complex patterns in radiographic images, including lung disease, breast cancer screening, and bone age estimation, and have outperformed traditional manual methods [10].

Recent AI methods have been applied to radiographic interpretation for skeletal maturity assessment, showing promise for categorizing vertebral maturation into clinically relevant phases such as early, peak, and late [7]. These advances suggest that AI may complement or even enhance conventional human assessment in CVM staging.

Specifically, machine learning models have been developed to classify cervical vertebral morphology, demonstrating high accuracy in predicting CVM stages and thus addressing the challenges of subjective manual assessment [11].

Previous studies provide promising evidence. Mohammad-Rahimi et al. [6] and Atici et al. [12] introduced fully automated CVM assessment systems that achieved high agreement with orthodontic experts and demonstrated that deep learning could reliably classify cervical vertebra maturation and pubertal growth spurts [10]. However, further studies are still needed to validate AI-based approaches against established human staging methods and to establish more objective, standardized approaches that yield consistent results across AI tools prior to their integration into standard clinical workflows.

Although AI-based models for cervical vertebra maturation have shown promising results, their diagnostic accuracy can vary, with reported accuracies ranging from 0.59 to 0.71 [7]. This variability underscores the importance of rigorous validation research to assess the reliability and generalizability of these models across a variety of clinical populations and imaging protocols [7]. Moreover, many models focus only on the six-stage CVM classification, which, while precise, is not always clinically actionable, and there is limited evidence regarding AI accuracy in simplified three-phase classifications (Early, Peak, Late) [10].

Recent evidence highlights both the potential and the limitations of AI-based skeletal maturity assessment. A 2025 systematic review and meta-analysis of 25 studies found generally promising but stage-dependent performance, with lower sensitivity for some intermediate CVM stages [7]. A subsequent meta-analysis of 21 studies reported a pooled accuracy of 0.83 for identifying the pubertal growth spurt but identified substantial heterogeneity, limited external validation, and methodological weaknesses [13]. Recent primary studies have also reported variable results. Chaves et al. found substantial agreement between an integrated YOLOv8–CNN model and human observers for CVM staging (κ = 0.786) [14], while Ramnarayan et al. reported an overall six-stage accuracy of 86% using a VGG19 model [15]. Yamada et al. further demonstrated that classification performance was strongly influenced by how the cervical region was presented to the network [16]. Collectively, these findings support the potential of AI-assisted CVM assessment while emphasizing the need for independent external validation.

The aim of this study was to determine the diagnostic accuracy of a fully automated deep-learning model, trained on the C2–C4 cervical vertebral region of lateral cephalometric radiographs, for classifying skeletal maturity into the six CVM stages (CS1–CS6) and into the corresponding three maturation phases (Early, Peak and Late), using calibrated expert staging as the reference standard [1,17]. Secondary outcomes were sensitivity, specificity and predictive values across growth phases.

## 2. Materials and Methods

### 2.1. Study Design and Setting

This study was designed as a cross-sectional diagnostic-accuracy and model-development study with internal cross-validation and external validation on an independent public benchmark dataset, aimed at developing and externally evaluating an automated deep-learning pipeline for cervical vertebral maturation (CVM) staging from full lateral cephalometric radiographs. The pipeline was designed as a three-stage cascade: (i) automatic detection of the cervical region containing the C2–C4 vertebrae [1,5], (ii) classification of the broader maturation phase (Early, Peak, or Late), and (iii) refinement to one of the six CVM stages (CS1–CS6) through a phase-specific binary classifier. Model development and internal cross-validation were performed exclusively on an institutional dataset; final test performance was assessed on the official test split of an independent public benchmark to evaluate generalizability across imaging centers.

Reporting standards. The study was reported primarily in accordance with the STARD 2015 guidelines for diagnostic-accuracy studies [18] and the CLAIM recommendations for artificial intelligence in medical imaging [19]. Relevant STROBE items were additionally considered when reporting the retrospective observational characteristics of the institutional cohort [20]. Completed STARD, CLAIM, and STROBE checklists are provided as Supplementary Materials.

### 2.2. Ethical Approval

The study was reviewed and approved by the Research Ethics Committee at the King Abdulaziz University Faculty of Dentistry, Jeddah, Saudi Arabia (reference number 106-06-25, approved on 4 December 2025). All cephalometric radiographs included in model development were retrieved from the institutional archive in fully anonymized form. No identifiable patient information was accessed at any stage of the study, and no new clinical data was collected.

### 2.3. Datasets

#### 2.3.1. Institutional Dataset (Training and Validation)

The institutional cohort comprised 523 lateral cephalometric radiographs obtained from patients aged 8–18 years who underwent routine orthodontic assessment at King Abdulaziz University Faculty of Dentistry and affiliated private orthodontic clinics from the year of 2023–2026. All eligible radiographs available during the predefined sampling period were consecutively included.

Age and recorded gender were extracted from the electronic patient records, while imaging information was obtained from the available DICOM metadata using a standardized data-extraction form. No patient-administered questionnaire was used. The radiographs had been acquired as part of routine orthodontic diagnosis and treatment planning using digital cephalometric imaging systems. Details of the imaging devices and acquisition parameters were recorded where available. The demographic characteristics of the institutional cohort are reported in Table 1, and its distribution across the six CVM stages and three maturation phases is presented in Supplementary Table S6.

#### 2.3.2. Inclusion and Exclusion Criteria

Inclusion criteria were (i) a lateral cephalometric radiograph obtained as part of routine orthodontic assessment; (ii) patient age between 8 and 18 years at the time of imaging; (iii) adequate visualization of C2–C4 for application of the CVM method [1,4,5]; and (iv) availability of a consensus CVM-stage label and the annotations required for the relevant model component.

Exclusion criteria were (i) congenital or acquired cervical spine abnormalities that could alter vertebral morphology; (ii) previous cervical trauma, surgery, or head-and-neck radiotherapy; (iii) systemic, endocrine, or syndromic conditions known to affect skeletal growth; (iv) inadequate visualization of C2–C4 because of poor image quality or superimposed artifacts; and (v) missing CVM-stage labels or incomplete annotations. These criteria were defined a priori to minimize factors that could interfere with CVM assessment or image analysis, consistent with the morphological requirements of the CVM method [1,4,5,21].

#### 2.3.3. Data Annotation and Reliability Assessment

Two trained and calibrated dentists independently assigned each radiograph to one of the six CVM stages, CS1–CS6, according to the established CVM method [1]. The six stages were subsequently grouped into three broader maturation phases: Early (CS1–CS2), Peak (CS3–CS4), and Late (CS5–CS6). Both examiners were blinded to patient demographic and clinical information and to each other’s assessments.

Before formal staging, the examiners completed a calibration exercise using 50 randomly selected lateral cephalometric radiographs. Inter-examiner and intra-examiner agreement were evaluated using Cohen’s kappa coefficient. Only examiners who achieved κ ≥ 0.80 for both assessments proceeded to formal staging. For intra-examiner reliability, a subset of the images was reassessed after a two-week interval. Reliability checks were repeated after every 50 annotated radiographs. Disagreements were resolved through consensus, with adjudication by an expert orthodontist when required.

In addition to the final CVM-stage labels, polygon annotations of C2, C3, and C4 were created to generate the reference bounding boxes used for detector training and region-of-interest extraction.

#### 2.3.4. Component-Specific Sample Sizes

The number of radiographs available for model development differed slightly across pipeline components because of component-specific annotation and preprocessing requirements. The original institutional cohort contained 523 CVM-labeled radiographs.

For the three-phase classifier, 520 radiographs were successfully loaded, and 517 yielded valid C2–C4 regions of interest; therefore, 517 images were included in five-fold cross-validation. For the phase-specific classifiers, 520 radiographs yielded valid regions of interest, comprising 69 Early-phase, 153 Peak-phase, and 298 Late-phase images. The YOLOv8 detector was also developed using 520 radiographs with valid bounding-box annotations.

Thus, 523 represents the original labeled institutional cohort, whereas the effective development sample differed according to the annotation and preprocessing requirements of each model component.

#### 2.3.5. Sample Size Consideration

No formal a priori sample-size calculation was performed. Sample-size requirements for machine-learning models in medical imaging depend on several factors, including model complexity, outcome distribution, image heterogeneity, and the intended validation strategy, and standardized approaches for determining the required development sample remain limited [22]. All eligible archival radiographs available during the predefined sampling period were consecutively included, yielding an institutional cohort of 523 images. This sample size was comparable to those used in previously published CVM deep-learning studies [6,23,24]; however, these comparisons are provided only to contextualize the available dataset and do not establish its statistical adequacy. External evaluation was performed using the complete 150-image official test split of the ‘Aariz benchmark [17]. At the observed overall accuracy of 96.0%, the corresponding 95% confidence interval was 92.7–98.7%. However, class-specific estimates were less precise because some external subgroups were small, particularly the Early phase (*n* = 10) and CS3 (*n* = 7). Accordingly, overall and class-specific results are reported with confidence intervals and should be interpreted in light of the limited subgroup sizes and class imbalance [18,22].

#### 2.3.6. Bias Control

Potential sources of selection, measurement, observer, and model-evaluation bias were considered. Selection bias was reduced by consecutively including all eligible archival radiographs available during the predefined sampling period, although the retrospective sampling frame and unequal stage distribution may have introduced spectrum bias.

Measurement bias related to image quality was reduced by applying predefined criteria for adequate visualization of C2–C4. Observer bias in the reference standard was addressed through examiner calibration, independent blinded staging, repeated reliability assessment, consensus resolution, and expert adjudication.

Optimism in internal performance estimates was limited by reporting out-of-fold (OOF) predictions from stratified five-fold cross-validation. The predefined ‘Aariz test split was reserved exclusively for external evaluation, with no external image used during model development, hyperparameter tuning, or model selection [18,19,20].

#### 2.3.7. ‘Aariz Benchmark Dataset (Independent External Test Set)

Final performance was evaluated on the official test split of the ‘Aariz dataset, a publicly available cephalometric benchmark introduced by Khalid et al. [17]. The full ‘Aariz dataset comprises 1000 lateral cephalometric radiographs collected from 1000 patients aged 8 to 62 years and acquired using seven distinct X-ray imaging devices with spatial resolutions ranging from 0.089 to 1.139 mm per pixel. To the best of our knowledge, ‘Aariz represents the first publicly available standard resource for CVM stage classification.

Each image is accompanied by 29 cephalometric landmark annotations and a single CVM stage label (CS1–CS6). Annotation was carried out in two phases by two trained dentists, who independently reviewed all CVM stage assignments; any disagreements were resolved by expert orthodontists. The final inter-rater agreement on the CVM stage between senior orthodontists was reported as 96.6% [17]. The dataset is provided in a pre-defined three-way split: 700 training images, 150 validation images, and 150 test images, with images from each X-ray machine distributed approximately uniformly across splits. For the present study, only the 150-image official test split was used; the training and validation partitions of ‘Aariz were not used during model development, hyperparameter tuning, or model selection, in order to preserve the integrity of the external evaluation.

The institutional dataset was used exclusively for model development, whereas the official 150-image ‘Aariz test split was reserved for independent external evaluation. The ‘Aariz training and validation partitions were not used because the study was designed to evaluate a model developed entirely outside the ‘Aariz dataset. Although use of the ‘Aariz training or validation partitions under a prespecified, locked protocol would not necessarily invalidate evaluation on its separate test split, excluding all ‘Aariz images from development provided a stricter assessment of performance across independent data sources [13,17].

#### 2.3.8. Image Preprocessing

**Figure 1:**
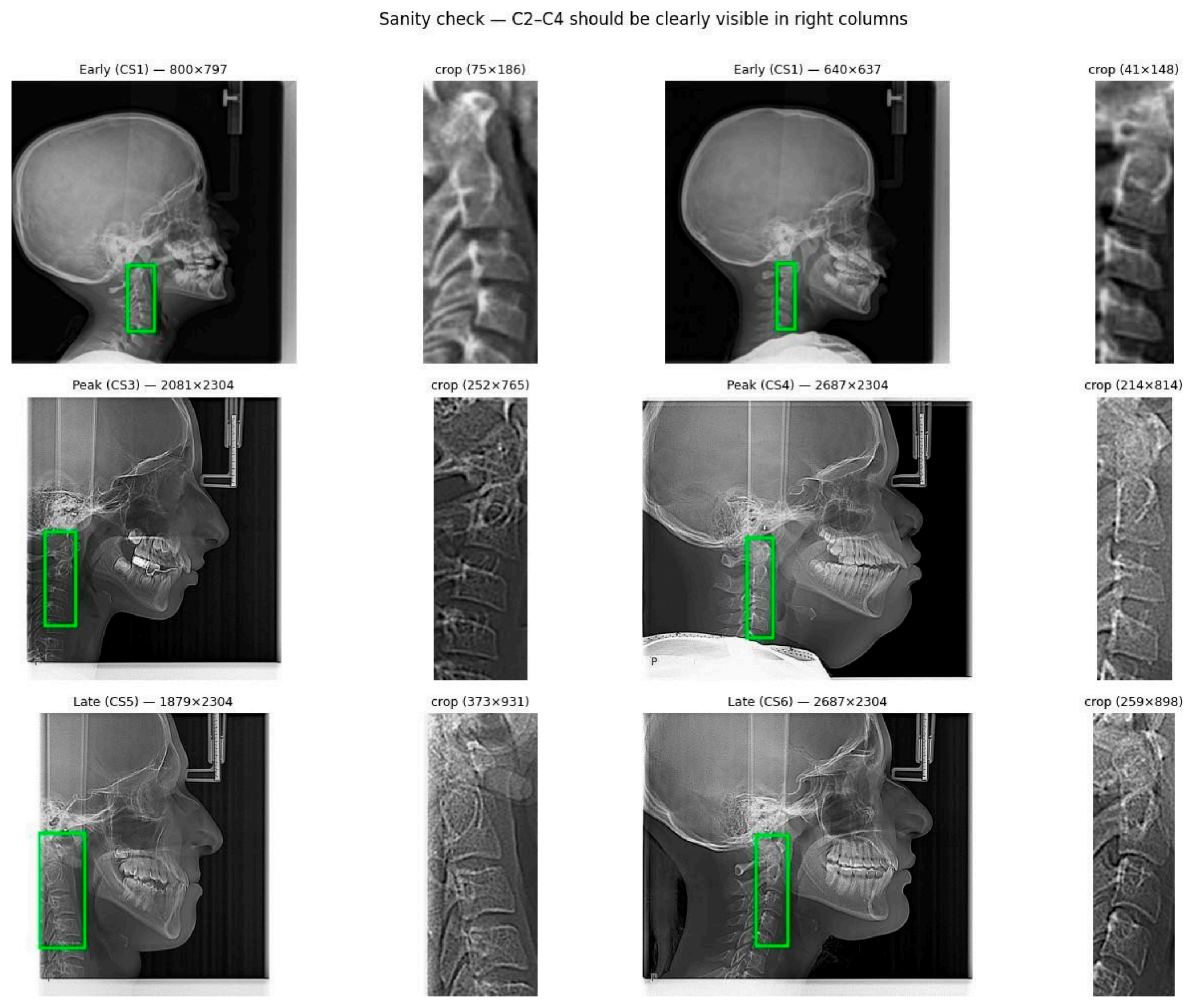
Representative cervical regions of interest. The green bounding box overlaid on the full cephalogram (**left**) is derived from the polygon annotations of the C2, C3, and C4 vertebrae. The corresponding CLAHE-enhanced crop (**right**) is the input passed to the classification models.

For each radiograph in the institutional dataset, the union of the three vertebral polygons was computed to obtain a tight bounding box encompassing the C2–C4 region. The box was expanded by 8% on each side to retain a small margin of contextual anatomy. Each radiograph was converted to grayscale, and Contrast-Limited Adaptive Histogram Equalization (CLAHE) was applied to improve local contrast while limiting noise amplification [25]. CLAHE was implemented with a clip limit of 2.0 and a tile-grid size of 8 × 8. The detected C2–C4 region was then cropped, resized to the input dimensions required by each model, and normalized using ImageNet mean and standard-deviation values for the ResNet-50 classifiers [26]. Representative crops are shown in Figure 1. For external test images, polygon annotations for the cervical vertebrae are unavailable; therefore, the YOLO detector described below was used to automatically extract the cervical region, thereby exactly replicating the deployment pipeline.

#### 2.3.9. Pipeline Overview

**Figure 2:**
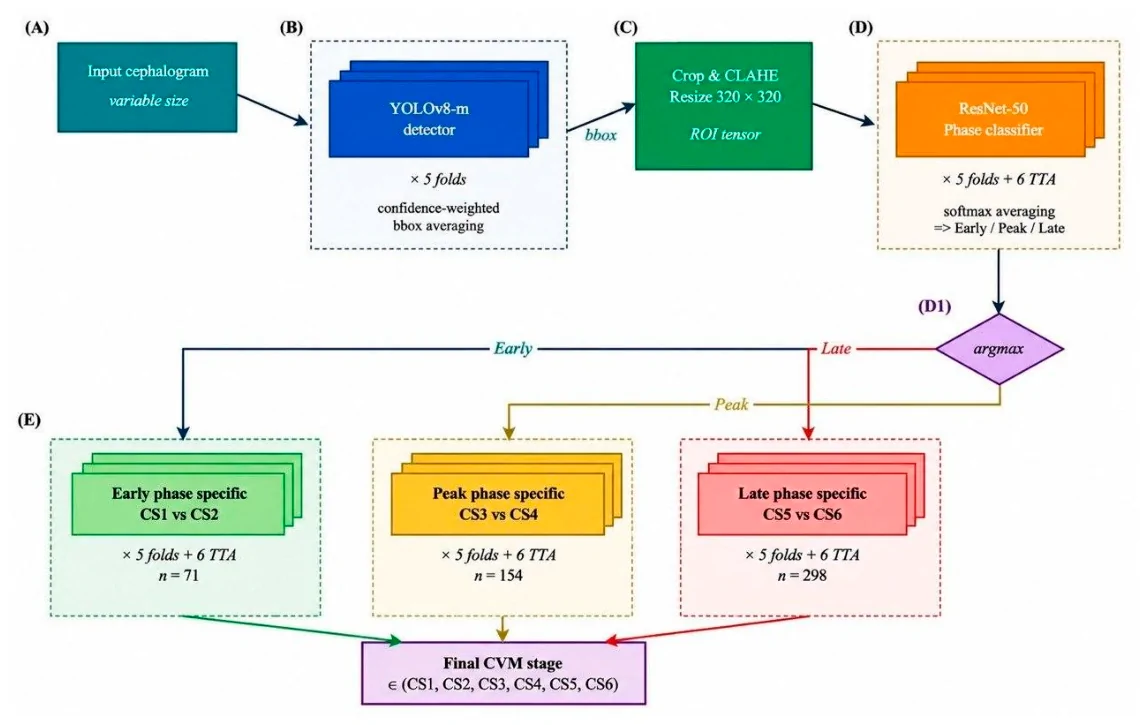
Overview of the proposed end-to-end pipeline. (**A**) A full lateral cephalometric radiograph of variable size is provided as input. (**B**) A YOLOv8-nano detector ensemble identifies the C2–C4 region [28]. (**C**) The detected region is cropped, contrast-enhanced using CLAHE, and resized to 320 × 320 pixels. (**D**) The processed region is passed to a ResNet-50 phase-classifier ensemble [26], which assigns the case to the Early, Peak, or Late maturation phase based on softmax probability averaging. (**D1**) An argmax decision selects the maturation phase with the highest averaged probability. (**E**) The predicted maturation phase determines the corresponding phase-specific binary classifier (CS1 vs. CS2, CS3 vs. CS4, or CS5 vs. CS6), which assigns the final six-stage CVM classification.

The proposed pipeline presented in Figure 2 consisted of three sequential components: (i) automated detection of the cervical region containing the C2–C4 vertebrae, which are used for CVM assessment [1,5]; (ii) classification into the broader maturation phases (Early, Peak, or Late) based on the established grouping of the six CVM stages [21,27]; and (iii) refinement to the corresponding CVM stage using a phase-specific binary classifier. The classification models used a ResNet-50 architecture pretrained on ImageNet [26]. Each module is implemented as an ensemble of five models trained under stratified five-fold cross-validation, the outputs of which are aggregated by softmax.

### 2.4. Cervical Region Detector

A YOLOv8-nano model (version 8.0.196) using the Ultralytics framework was used to detect the C2–C4 region [28]. The single-class formulation reflected the goal of localizing one anatomically consistent region per image, while the lightweight architecture balances inference speed with accuracy. The polygon-derived bounding boxes were converted to YOLO format (normalized center coordinates and dimensions) and used as ground truth.

Stratified five-fold cross-validation was applied to estimate generalization [22]. Each fold was trained for up to 80 epochs at an input resolution of 640 × 640 pixels, with early stopping on the validation mAP@0.5 (patience = 15). Standard YOLO augmentation was used, with horizontal flipping disabled because cervical vertebral concavities are direction-dependent.

### 2.5. Phase Classifier (Early, Peak, Late)

A ResNet-50 backbone, pretrained on ImageNet [26], was used as the feature extractor for the phase classifier, with a single linear classification head producing three logits [29]. Training used a batch size of 16, an input resolution of 320 × 320 pixels, the AdamW optimizer with learning rate 1 × 10^−4^ and weight decay 1 × 10^−4^, a cosine-annealing learning-rate schedule, label-smoothed cross-entropy loss (smoothing = 0.1), and dropout of 0.2 prior to the classification head.

To address the substantial class imbalance, a WeightedRandomSampler was used during training so that all three phases were drawn with equal expected frequency per epoch. Each draw was paired with an independently sampled augmentation from a mild on-the-fly pipeline (rotation ±6°, translation ±4%, scale ±6%, brightness/contrast ±10%, light Gaussian noise; horizontal flipping disabled). Training proceeded for up to 50 epochs with early stopping on the validation macro-F1 (patience = 10). The classifier was evaluated using stratified five-fold cross-validation on the institutional dataset. At inference, six-view test-time augmentation (TTA) was applied, and the resulting softmax distributions were averaged.

### 2.6. Phase-Specific Binary Classifiers

Three binary classifiers were trained, one per phase, using radiographs that yielded valid C2–C4 regions of interest after preprocessing: an Early phase-specific classifier (CS1 vs. CS2; *n* = 69), a Peak phase-specific classifier (CS3 vs. CS4; *n* = 153), and a Late phase-specific classifier (CS5 vs. CS6; *n* = 298). Although the original labelled cohort contained 71 Early-phase and 154 Peak-phase radiographs, two CS1 images and one CS3 image did not yield valid regions of interest and were excluded from specialist-model development. The final specialist datasets therefore comprised 35 CS1 and 34 CS2 images for the Early classifier and 41 CS3 and 112 CS4 images for the Peak classifier. These effective sample sizes are those reported in Supplementary Table S9.

The specialist stage used a DenseNet121 backbone, pretrained on ImageNet—a different architecture from the ResNet-50 phase classifier by design. The cascade reduces the classification problem in two steps: the phase classifier first narrows the six candidate CVM stages down to the three broader maturation phases (Early, Peak, Late), and the corresponding phase-specific binary classifier then resolves the remaining two-way distinction within that phase (e.g., CS1 vs. CS2 for an image routed to Early). Because the two stages use different backbone architectures, no weights are shared or transferred between them; each specialist was trained independently within its phase-specific dataset. As a result, no validation image could contribute to a specialist model through shared phase-classifier weights, eliminating any potential source of cross-validation leakage between the two stages.

### 2.7. Evaluation Strategy

Model development used internal stratified five-fold cross-validation on the institutional dataset. Within each fold, approximately 80% of the data served as the training partition and the remaining 20% as the validation partition. For the specialist classifiers, this independence held for both the specialist training folds and model initialization, as no phase-classifier weights were transferred in the reported experiments. The OOF protocol provides an honest estimate of generalization within the source distribution. Final test performance was obtained on the 150-image official test split of the ‘Aariz benchmark. The full pipeline was applied end-to-end: the YOLO detector ensemble produced an automatic bounding box for each test image; the cropped region was passed to the phase classifier ensemble; and the predicted phase determined the corresponding specialist ensemble. No ‘Aariz data were used during model development at any stage [17,18,19].

### 2.8. Evaluation Metrics

For the detector, mean Average Precision at IoU = 0.5 (mAP@0.5), mAP@0.5:0.95, precision, recall, and per-image Intersection over Union (IoU) against the polygon-derived bounding boxes were reported. For the classifiers, accuracy, macro-averaged F1, Cohen’s κ, quadratic-weighted κ, and macro-averaged ROC-AUC were reported. The quadratic-weighted κ is particularly relevant in this context because it penalizes misclassifications between adjacent CVM stages less heavily than between distant ones, consistent with the ordinal nature of the staging task.

Six-stage class probabilities and ROC-AUC for the hierarchical cascade. Because the cascade assigns the final CVM stage in two steps, a six-stage probability vector was derived for each external test image by combining the ensemble outputs hierarchically. The phase-classifier ensemble provided the softmax probabilities of the Early, Peak, and Late phases, and each phase-specific ensemble provided the probabilities of the two stages within its phase. The probability of each CVM stage was computed as the product of the probability of its parent phase and the within-phase probability of that stage (for example, P(CS3) = P(Peak) × P(CS3|Peak)). Because the two within-phase probabilities sum to one, the six resulting values sum to one and form a valid probability distribution over CS1–CS6; the composite confidence score described in the Results is the product of the selected phase and specialist probabilities, i.e., the entry of this vector corresponding to the predicted stage. The macro-averaged ROC-AUC of the six-stage cascade was then calculated from this probability vector in a one-versus-rest manner, averaging the six per-stage AUCs with equal weight, using the same procedure as for the three-phase classifier. The hard six-stage prediction used for accuracy, F1, and κ was the stage selected by the specialist corresponding to the predicted phase, as described above.

Confidence intervals. The 95% confidence intervals reported for all external metrics were obtained by non-parametric bootstrapping with 1000 resamples. In each resample, the images of the relevant evaluation set (all 150 test images for the phase classifier and the end-to-end cascade; the 10, 47, and 93 images of the parent phase for the Early, Peak, and Late phase-specific classifiers, respectively) were drawn with replacement, keeping each image’s reference label, predicted label, and predicted probabilities together, and every metric was recomputed on the resampled set. The 2.5th and 97.5th percentiles of the 1000 bootstrap estimates were reported as the lower and upper confidence limits (percentile method). Resampling was performed on images without stratification by class, so that the sampling variability of the small classes was reflected directly in the interval width; this is why the intervals for the Early phase-specific classifier (*n* = 10) and for CS3 (*n* = 7) are very wide. In resamples in which a class was absent or had no predicted instances, the corresponding per-class term was undefined and was omitted from the macro-average for that resample, and for ROC-AUC, a resample in which a class had no positive or no negative instances was discarded and redrawn. Because percentile bootstrap intervals derived from very small subsets are approximate and may not attain nominal coverage, the intervals for these subsets should be regarded as indicative rather than exact [30].

### 2.9. Software and Hardware

All experiments were performed on the Kaggle cloud platform using a single NVIDIA Tesla T4 GPU (16 GB VRAM). The implementation used PyTorch (version 3.13.0), the timm library for the ResNet-50 backbone, Ultralytics YOLOv8 for object detection, Albumentations for image augmentation, OpenCV for image processing, and scikit-learn for evaluation metrics and stratified cross-validation splitting.

### 2.10. Statistical and Diagnostic-Accuracy Analysis

Data were analyzed with R statistical software (version 4.5.0). Demographic and clinical data were summarized using descriptive statistics: mean and standard deviation (SD) were reported for continuous variables, while categorical variables were presented as frequencies and percentages. Both examiners independently assessed each radiograph without being aware of the other’s ratings; when there was disagreement, the final CVM stage was determined by discussion between the two examiners.

Complementary statistics were used to assess agreement between the two raters. The simple percentage agreement was the proportion of cases in which the two raters agreed on the CVM stage. Cohen’s kappa (κ) was calculated in three versions (unweighted, linear-weighted, and quadratic-weighted), since CVM is an ordinal measure and weighted versions give partial credit for near-misses between stages. Gwet’s AC1 coefficient was calculated as an alternative to Cohen’s kappa because kappa is known to be sensitive to prevalence bias when the category distribution is imbalanced.

Krippendorff’s alpha (α) was computed under both nominal and ordinal assumptions, and the intraclass correlation coefficient (ICC) was calculated with a two-way agreement model for single measures. Interpretation of agreement coefficients was based on the guidelines of Landis and Koch, in which values greater than 0.80 are interpreted as almost perfect agreement. All tests were conducted at a significance level of α = 0.05.

## 3. Results

The institutional dataset comprised 523 labeled cephalometric radiographs. The effective sample size differed slightly among model components because of image availability and preprocessing requirements. For the three-phase classifier, 520 radiographs were successfully loaded, and 517 yielded a valid C2–C4 region of interest, resulting in 517 images used for five-fold internal cross-validation. For the phase-specific sub-stage classifiers, 523 radiographs were initially available, and 520 yielded valid regions of interest; these comprised 69 Early-phase, 153 Peak-phase, and 298 Late-phase images. The YOLOv8 detector was likewise developed using 520 radiographs with valid bounding-box annotations. Thus, the original cohort size was 523, whereas the effective training and validation sample size depended on the requirements of each model component.

Each model component underwent stratified five-fold cross-validation using its corresponding eligible dataset. Because the effective sample sizes differed across components, the exact numbers of training and validation images varied slightly between folds. For the final test assessment, all 150 cephalograms of the ‘Aariz test split were processed through the full end-to-end pipeline.

The mean patient age was 13.7 ± 2.9 years, indicating a predominantly adolescent cohort during the peak growth period (Table 1). Gender bias was low, with an almost equal distribution of females (51%) and males (49%). All images were of high quality, ruling out measurement bias due to poor quality. In terms of skeletal maturation, most patients were in the later developmental stages, with a high proportion in CS5 (42.3%) and CS4 (21.4%) and a lower proportion in CS1 (7.1%) and CS2 (6.5%). When categorized by growth phase, more than half of the sample (57.0%) were in the late phase, 29.4% in the peak phase, and 13.6% in the early phase. This indicates that the sample is biased toward those past the peak pubertal growth phase, which could affect the interpretation of developmental timing and treatment outcome.

Data were extracted retrospectively from archival electronic patient records and DICOM headers using a standardized data-extraction form; no patient-administered questionnaire was used. Image quality was graded independently by two calibrated examiners using predefined written criteria [18,19]. Percentages were calculated on the full institutional dataset (*n* = 523).

The inter-rater reliability analysis showed very high agreement between the two raters. The percentage agreement was 98.9%, indicating almost perfect agreement. Cohen’s kappa coefficients ranged between 0.984 (unweighted) and 0.989 (quadratic-weighted), all *p* < 0.001, indicating agreement much greater than chance. Likewise, Gwet’s AC1 statistic (0.987; CI = 0.98, 1.00) and Krippendorff’s alpha (0.994 for ordinal and 0.985 for nominal data) supported this conclusion. The intraclass correlation coefficient (ICC = 0.989; CI = 0.99–0.99) also showed excellent agreement. All values fall in the almost-perfect category, showing that CVM staging was highly consistent and reliable. The complete inter-rater agreement statistics are reported in Supplementary Table S7, and the inter-rater confusion matrix is shown in Supplementary Figure S2.

Despite substantial overall agreement, some disagreements occurred; these were few and usually reflected a difference of one CVM stage, suggesting slight interpretative variability. One exception (CVM-332) showed a larger disagreement of four stages (CS5 vs. CS1), which may represent an error or an ambiguous radiograph. The low frequency and minimal severity of disagreements further indicate the high quality of the rating procedure. The individual cases of annotator disagreement, together with the adjudicated CVM stage and the stage distance for each, are listed in Supplementary Table S8. The mean OOF IoU between predicted and ground-truth bounding boxes was 0.8884, with 97.9% of images exceeding IoU = 0.70 and 81.3% exceeding IoU = 0.85; the detector produced no failed predictions. A summary of the YOLO detector performance is presented in Supplementary Figure S3.

The detector achieved excellent internal cross-validation performance (mean mAP@0.5 0.994 ± 0.002; Supplementary Table S1). The internal cross-validation performance of the phase classifier is summarized in Supplementary Table S2, and the cross-validation summaries for all three phase-specific sub-stage specialists are consolidated in Supplementary Table S9. The corresponding per-fold cross-validation performance of the Early, Peak, and Late phase-specific classifiers is detailed in Supplementary Tables S3–S5. The three-phase classifier achieved an OOF accuracy of 77.4%, with a macro F1 of 0.699, Cohen’s κ of 0.608, and quadratic-weighted κ of 0.762. Among the phase-specific classifiers, the Peak specialist achieved a mean cross-validation AUC of 0.617 ± 0.150 and an OOF AUC of 0.619.

The external test set comprised 150 lateral cephalometric radiographs drawn from the official test split of the ‘Aariz benchmark. The dataset reflects the natural class imbalance observed in orthodontic practice, with Late-phase images (CS5–CS6) constituting the majority (*n* = 93, 62.0%), Peak-phase images a moderate proportion (*n* = 47, 31.3%), and Early-phase images the smallest group (*n* = 10, 6.7%). Within the Late phase, CS5 was substantially more frequent than CS6 (69 vs. 24 images), consistent with the broader epidemiological patterns reported in the original ‘Aariz publication. The full composition of the external test set, broken down by phase and stage, is provided in Supplementary Table S10.

The YOLOv8-nano detector ensemble produced a bounding box for each of the 150 external test images, with no inference failures. The median relative bounding-box area was 0.043 (interquartile range: 0.035–0.052). Because reference C2–C4 bounding boxes were not available for the external dataset, quantitative localization accuracy, including a formal localization success rate, could not be established. Accordingly, the external detector results are reported descriptively in terms of successful box generation and bounding-box size rather than as a validated 100% localization rate. The distribution of relative bounding-box areas across the external test set is shown in Supplementary Figure S4.

**Figure 3:**
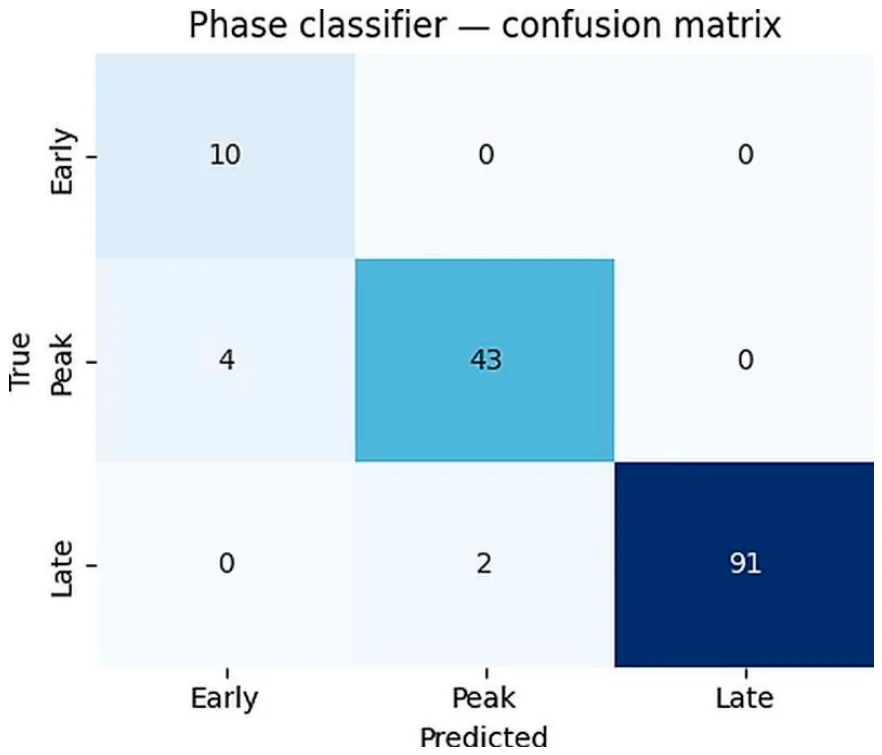
Confusion matrix of the phase classifier on the external test set (*n* = 150). Rows correspond to the reference phase assigned by expert consensus and columns to the phase predicted by the model, so diagonal cells contain correctly classified radiographs and off-diagonal cells misclassifications. Cell entries are absolute numbers of radiographs and color intensity scales with cell count. Reference class sizes were Early *n* = 10, Peak *n* = 47 and Late *n* = 93; off-diagonal counts should therefore be read relative to these denominators rather than compared directly with one another.

The three-phase classifier achieved an accuracy of 96.00% [95% CI: 92.67%, 98.67%] on the external test set, with a macro F1 of 0.9191 [95% CI: 0.8302, 0.9745], Cohen’s κ of 0.9236 [95% CI: 0.8591, 0.9742], and quadratic-weighted κ of 0.9511 [95% CI: 0.9079, 0.9836]. The macro ROC-AUC was 0.9975 [95% CI: 0.9935, 0.9998]. The full metric table with confidence intervals and per-class clinical metrics is presented in Table 2A–C. The confusion matrix is shown in Figure 3.

The Early phase was classified with perfect sensitivity (1.0000), with four false positives in which Peak images were incorrectly assigned to the Early phase. The Late phase was classified with perfect precision (PPV = 1.0000) and near-perfect sensitivity (0.9785). The Peak phase showed the weakest performance, with four missed Peak cases reassigned to the Late phase, reflecting the known morphological similarity between the later stages of Peak and the early stages of Late maturation.

The three binary phase-specific classifiers were evaluated on the images whose true phase matched the specialist’s parent phase. The corresponding confusion matrices for the Early, Peak, and Late phase-specific classifiers are shown in Supplementary Figure S5.

Early phase-specific (CS1 vs. CS2). Evaluated on the 10 Early-phase images (nCS1 = 5, nCS2 = 5), it achieved 80.00% accuracy [95% CI: 50.00%, 100.00%] with a macro F1 of 0.7917 [95% CI: 0.4444, 1.0000] and Cohen’s κ of 0.6000 [95% CI: 0.0909, 1.0000]. The AUC was 1.0000; however, with only 10 Early-phase images (five per stage), this value should be regarded as an imprecise estimate rather than as evidence of a well-separated decision boundary. CS2 was classified with perfect sensitivity (1.0000), while CS1 had a sensitivity of 0.6000, with two CS1 images misclassified as CS2. The wide confidence intervals reflect the small sample size. The full metric table for the Early phase-specific classifier is provided in Supplementary Table S11. Peak phase-specific (CS3 vs. CS4). Evaluated on 47 images (nCS3 = 7, nCS4 = 40), it achieved 97.87% accuracy [95% CI: 93.62%, 100.00%] with a macro F1 of 0.9603 [95% CI: 0.8626, 1.0000] and Cohen’s κ of 0.9207 [95% CI: 0.7283, 1.0000]. The AUC was 1.0000.

This perfect AUC should be interpreted cautiously given the small and imbalanced external Peak subset (*n* = 47; CS3 = 7, CS4 = 40). CS3 was classified with perfect sensitivity (1.0000), while one CS4 image was misclassified as CS3. The full metric table for the Peak phase-specific classifier is provided in Supplementary Table S12.

Late phase-specific (CS5 vs. CS6). Evaluated on 93 images (nCS5 = 69, nCS6 = 24), it achieved 76.34% accuracy [95% CI: 67.74%, 84.95%] with a macro F1 of 0.7244 [95% CI: 0.6246, 0.8220] and Cohen’s κ of 0.4561 [95% CI: 0.2573, 0.6467]. The macro AUC was 0.8062 [95% CI: 0.6747, 0.9315]. Both CS5 and CS6 achieved a sensitivity of 0.7500, with 16 CS5 images misclassified as CS6 and 6 CS6 images misclassified as CS5. The late-phase-specific classifier showed the weakest performance among the three, consistent with the known difficulty of the CS5–CS6 distinction and the moderate class imbalance (CS5:CS6 = 2.9:1) in this test subset. The full metric table for the Late phase-specific classifier is provided in Supplementary Table S13.

**Figure 4:**
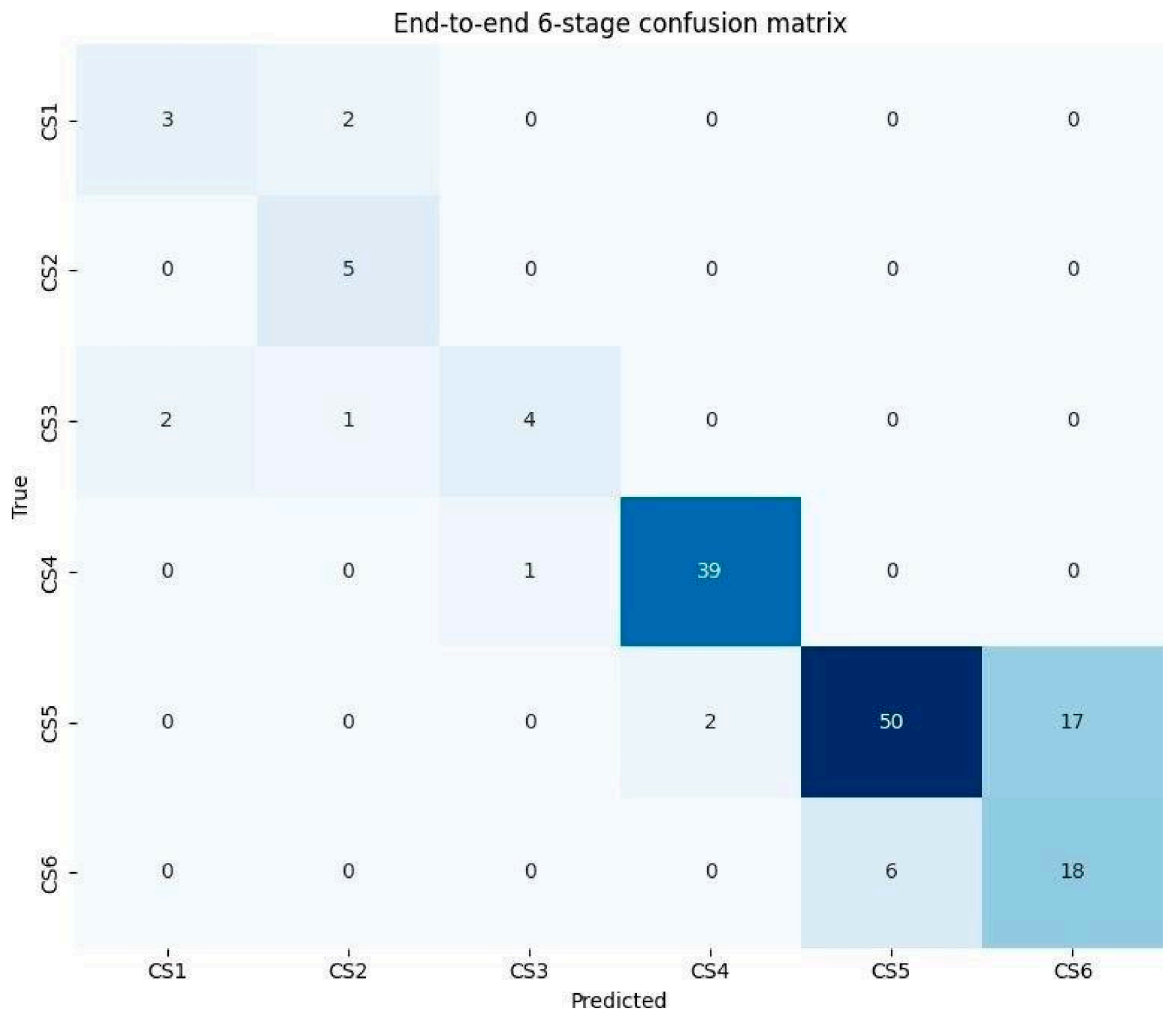
Confusion matrix for the end-to-end six-stage CVM classification on the external test set (*n* = 150). Rows represent the reference CVM stages assigned by expert consensus, and columns represent the stages predicted by the fully automated cascade. Diagonal cells indicate correct classifications, whereas off-diagonal cells indicate misclassifications. Cell values represent the absolute number of radiographs. Because each phase-specific classifier can predict only one of the two CVM stages within the phase selected by the upstream phase classifier, mapping the final six-stage predictions back to their corresponding phases necessarily reproduces the original phase-classifier predictions. Accordingly, the phase-level performance of the end-to-end cascade was identical to that of the standalone phase classifier: accuracy, 96.0%; macro F1, 0.9191; Cohen’s κ, 0.9236; quadratic-weighted κ, 0.9511; and macro ROC-AUC, 0.9975. The previously reported cascade-derived phase accuracy of 96.67% resulted from an inconsistency in the prediction summary and was corrected to 96.0%. The corresponding phase-level results are presented in Table 3.

The full end-to-end cascade was applied to all 150 test images, with the cervical region automatically detected by the YOLO ensemble; each image was routed through the phase classifier and the corresponding sub-stage specialist without manual intervention. Metrics are reported in Table 2C, and the six-stage confusion matrix is shown in Figure 4. Per-class clinical metrics (sensitivity, specificity, PPV, and NPV) for the end-to-end six-stage cascade are provided in Supplementary Table S14.

**Table 3:** Phase- and stage-level correctness of the end-to-end cascade on the external test set (*n* = 150).

Phase	*n*	Phase Correct (%)	Stage Correct (%)
Early	10	10 (100.0%)	8 (80.0%)
Peak	47	43 (91.5%)	43 (91.5%)
Late	93	91 (97.8%)	68 (73.1%)
Overall	150	144 (96.0%)	119 (79.3%)

Of the 150 test images, 119 (79.3%) were correctly classified at the six-stage level. Among the 31 misclassifications, 29 (19.3% of all images) were off-by-one errors—that is, the predicted stage was immediately adjacent to the true stage—and only 2 (1.3%) were off by two or more stages. Consequently, 98.7% of all predictions were within ±1 stage of the true label (Table 4). The predominance of adjacent-stage errors indicates that most misclassifications occurred between neighboring CVM categories rather than between widely separated stages. However, adjacency alone does not establish that an error is clinically inconsequential, because misclassification between neighboring stages may still influence the interpretation of skeletal maturation and the timing of growth-related treatment decisions.

Stage-level accuracy ranged from 0.571 (CS3) to 1.000 (CS2). The lowest accuracies were observed for CS3 (4 of 7, 0.571) and CS5 (50 of 69, 0.725). CS4 showed near-perfect accuracy (39/40, 0.975), consistent with the largest per-stage sample size in the test set. At the phase level, correctness was 100.0% for Early (10/10), 91.5% for Peak (43/47), and 97.8% for Late (91/93), corresponding to an overall phase-classification accuracy of 96.0% (144/150). Stage-level accuracy was lower, particularly in the Late phase (73.1%), primarily because of CS5–CS6 confusion.

The dominant source of error was CS5 → CS6 confusion (17 cases, 54.8% of all errors), followed by CS6 → CS5 (6 cases). Together, these two adjacent-stage confusions within the Late phase accounted for 74.2% of all misclassifications. All remaining confusion patterns involved only one or two cases and were also between adjacent stages, confirming the predominantly off-by-one character of the error distribution. Although these errors were predominantly adjacent in ordinal stage, the high concentration of errors at the CS5–CS6 boundary warrants caution in clinical interpretation. Depending on the treatment context, an adjacent-stage misclassification may still affect assessment of maturation status and treatment timing; therefore, these errors should not be regarded uniformly as clinically mild.

An exploratory composite confidence score was calculated as the product of the phase-classifier confidence and the selected specialist-classifier confidence. This product was used only as a descriptive measure of model certainty and should not be interpreted as a calibrated probability [31]. The mean composite confidence score was 0.532 for correct predictions and 0.476 for incorrect predictions. When predictions were grouped by composite-confidence intervals (Supplementary Figure S1), higher-confidence bins generally corresponded to higher empirical accuracy, and the lowest-confidence bin (mean confidence ≈ 0.25, *n* = 5) also showed low empirical accuracy (0.20), suggesting that the pipeline’s uncertainty in that region was appropriately reflected in low confidence scores; however, no formal probability-calibration procedure or calibration metric was applied.

The ten incorrect predictions with the highest composite confidence scores (0.549–0.705) were all CS5 images misclassified as CS6. This concentration of relatively confident errors in a single confusion pair (CS5 → CS6) suggests that the Late phase-specific classifier systematically over-predicts CS6 for a subset of CS5 images, likely reflecting the morphological overlap between the two stages. Conversely, the ten incorrect predictions with the lowest composite confidence scores (0.259–0.395) were distributed across multiple confusion pairs, indicating that the pipeline’s uncertainty was appropriately elevated for these more ambiguous cases (Supplementary Tables S15 and S16). These findings are descriptive and exploratory and do not establish formal probability calibration.

## 4. Discussion

The proposed model demonstrated strong performance for broad three-phase classification but lower accuracy for detailed six-stage CVM classification. Although the Late phase was identified with high phase-level accuracy, the Late phase-specific classifier showed the weakest stage-level performance, primarily because of confusion between CS5 and CS6. This distinction indicates that reliable assignment to a broader maturation phase does not necessarily imply equally reliable discrimination between its constituent CVM stages [4,5]. Therefore, phase-level and stage-level performance should be interpreted separately, particularly when the predicted stage may influence the timing of growth-related orthodontic treatment.

External performance exceeded the internal cross-validation estimates (three-phase accuracy 77.4% out-of-fold versus 96.0% on the external test set). This direction of change is also atypical: in a systematic review of externally validated deep-learning algorithms for radiologic diagnosis, 81% of algorithms performed worse, not better, on the external dataset than internally [32]. Several factors may have contributed to this discrepancy, including differences in sample size, class distribution, imaging acquisition, population characteristics, and case composition between the institutional and ‘Aariz datasets [33]. In particular, the external Peak subset contained only 47 cases, of which only 7 were CS3, and the external Early subset contained only 10 cases, so the perfect external AUCs of these two specialists should be interpreted cautiously because of the limited and imbalanced sample; performance estimates derived from samples of this size carry wide confidence intervals, and the error bars around cross-validated estimates are routinely underestimated at such sample sizes [30]. The unexpectedly high external results may therefore reflect sampling variability and dataset-specific composition rather than a true improvement in generalization.

Overall, the findings suggest that automated CVM assessment is clinically feasible at the maturation-phase level, although precise sub-stage classification remains challenging.

The excellent performance of the detection module may be explained by the relatively consistent anatomical location of the C2–C4 vertebrae and the use of high-quality polygon annotations during training. In contrast, the lower classification performance, particularly for six-stage classification, was likely influenced by several factors. First, the dataset showed substantial class imbalance, with fewer Early-stage than Late-stage samples, which likely biased the model toward dominant classes and reduced sensitivity for early maturation stages. Second, adjacent CVM stages share subtle morphological differences, making fine-grained discrimination inherently difficult [4,5]; most classification errors occurred between neighboring stages rather than distant ones, supporting this explanation. The relatively small dataset size for deep learning, particularly for specialist classifiers, may also have limited performance [22]. In addition, the cascade architecture introduced error propagation, whereby incorrect phase classification led to downstream stage-classification errors. From a clinical perspective, phase-level classification may still be sufficient for orthodontic treatment timing and growth assessment [1]; therefore, the proposed system may have greater value as a screening or decision-support tool than as a fully autonomous diagnostic system [33].

Although the model demonstrates acceptable statistical performance for three-phase classification, this does not fully translate into reliable clinical decision-making at the six-stage level. Clinically, phase-level classification is often sufficient for determining treatment timing; therefore, despite limitations in six-stage classification, the model may still provide meaningful clinical utility as a screening or decision-support tool. However, its current performance does not support autonomous diagnostic use.

The decision to decompose the six-stage classification problem into a phase classifier followed by three binary classifiers was based on two considerations. First, classification into three broader maturation phases is less complex than direct six-stage classification because adjacent stages within the same phase share similar morphological features and differ only in subtle characteristics. Second, the hierarchical design allows each phase-specific classifier to focus on the distinction between the two stages within a single maturation phase [12,34]. In the present implementation, the phase-specific classifiers were initialized independently using ImageNet-pretrained weights rather than weights transferred from the phase classifier [26]. Therefore, their internal cross-validation results reflect independent phase-specific training without transferred upstream weights.

The present findings are broadly consistent with the existing literature on AI-based CVM assessment and highlight important methodological considerations that influence model performance. The moderate accuracy achieved for six-stage classification is comparable to previously reported fully automated deep-learning approaches. For example, Mohammad-Rahimi et al. [6] reported an accuracy of 61.6% for six-stage classification using 890 lateral cephalograms, with performance increasing substantially to 82.8% when the task was simplified to three maturation phases. Similarly, Li et al. [27] achieved six-stage accuracies of 67.1% and 70.4% using considerably larger datasets of 6079 and 10,200 cephalograms, respectively. Collectively, these findings reinforce the well-established observation that coarse-grained classification consistently outperforms fine-grained six-stage classification, because adjacent CVM stages exhibit subtle morphological differences that are inherently more difficult for deep-learning models to distinguish [6]. Moreover, the progressive improvements reported in studies using substantially larger datasets support the growing consensus that accurate stage-level CVM classification is highly dependent on dataset size and diversity, enabling models to better capture the considerable biological variability across skeletal maturation stages [35].

Nevertheless, several studies—for example, those of Zhou et al. [23], Radwan et al. [24] and Akay et al. [36]—have reported substantially higher diagnostic performance than that observed here. These differences should be interpreted cautiously, because many such approaches rely on fundamentally different methodological frameworks rather than representing direct comparisons. Landmark-based or semi-automated systems frequently incorporate manually identified cephalometric landmarks or other forms of expert-guided preprocessing before classification, thereby reducing the complexity of the learning task and minimizing anatomical variability presented to the model [37]. Additionally, many of these studies employed larger datasets collected under highly standardized imaging conditions, both of which are recognized contributors to improved model performance [38].

A notable methodological distinction of the present study is its fully automated, end-to-end pipeline, which performs CVM assessment directly from raw lateral cephalometric radiographs without requiring manual landmark identification or expert preprocessing. This design differs fundamentally from landmark-based artificial neural network approaches, such as that reported by Amasya et al. [11], who achieved excellent agreement (κ = 0.926) using manually placed cephalometric landmarks as model inputs. Although such approaches demonstrate high diagnostic performance, they remain dependent on expert orthodontists to accurately identify anatomical landmarks before prediction, thereby limiting automation and increasing operator dependency. In contrast, the current framework eliminates this manual step, providing a fully automated workflow that is more compatible with large-scale clinical implementation and routine orthodontic practice. Consequently, while the achieved classification accuracy may be modestly lower than that reported for landmark-assisted systems, the proposed approach offers substantially greater scalability, reproducibility, and potential for real-world clinical deployment, representing an important step toward practical AI-assisted skeletal maturity assessment [11].

## 5. Limitations

This study has several limitations. First, model development relied on a retrospective convenience sample of 523 radiographs obtained from King Abdulaziz University Faculty of Dentistry and affiliated private orthodontic clinics, and no formal a priori sample-size calculation was feasible for a deep-learning model [22]; the resulting class imbalance, with only 71 images in the Early phase, limited training of the CS1-versus-CS2 specialist and widened the confidence intervals around its performance. Second, the reference standard was consensus staging by two calibrated examiners rather than an independent biological gold standard such as hand–wrist maturation or statural growth velocity, so the model’s accuracy is bounded by the reproducibility of the CVM method itself, which has been questioned [4,21]. Third, external validation used a single public benchmark of 150 images [17]; although these were acquired on seven different devices, they do not represent the full range of populations, ethnicities, age structures, and acquisition protocols encountered in practice, and no second external cohort was available. Fourth, the design was cross-sectional and retrospective, so no inference can be drawn about the effect of the model on treatment-timing decisions or on clinical outcomes. Fifth, the pipeline was not compared prospectively with clinicians working under routine conditions, and reading time, workflow integration and clinician–model interaction were not assessed. Finally, subgroup analyses by gender and by age band were not pre-specified and were not adequately powered, and model calibration was assessed only descriptively.

Two further constraints deserve emphasis because they directly influence how the accuracy reported here should be read. First, the development and test sets do not share an age spectrum: the institutional dataset was restricted to patients aged 8–18 years, whereas the ‘Aariz benchmark spans 8–62 years [17]. Cervical vertebral morphology continues to change after skeletal maturity, so a proportion of the external test images lie outside the age range on which the model was trained. This domain shift is a plausible contributor to the concentration of errors in the CS5–CS6 confusion pair, and it means the six-stage accuracy reported here should not be extrapolated to adult populations. Second, neither dataset is large by deep-learning standards: 523 development and 150 external test radiographs distributed across six ordinal stages and two genders leave several cells with fewer than ten observations, which is why gender-stratified and age-stratified accuracy estimates were not reported—they would have been too imprecise to interpret. Adequately powered, prospectively recruited, multicentre datasets with balanced representation of stage, gender and age are therefore required before clinical deployment [14].

## 6. Conclusions

This study developed and evaluated a fully automated deep-learning pipeline for CVM assessment from lateral cephalometric radiographs. On one independent external benchmark, the model demonstrated promising performance for broad three-phase classification and moderate performance for detailed six-stage classification. However, the substantial difference between internal and external estimates, class imbalance, small external subgroups, and reliance on a single external dataset preclude firm conclusions regarding generalizability or clinical utility. Further multicenter prospective validation and comparison with clinicians under routine practice conditions are required before clinical implementation.

## Figures and Tables

**Table 1:** Distribution of patient demographics and clinical characteristics in the original institutional cohort (*n* = 523).

Characteristic	Frequency (%)
**Age (years) (Mean ± SD), No. of radiographs**	13.7 ± 2.9, 523
Gender—Female	266 (51%)
Gender—Male	257 (49%)
Image quality—Excellent	523 (100%)
CVM Stage—CS1	37 (7.1)
CVM Stage—CS2	34 (6.5)
CVM Stage—CS3	42 (8.0)
CVM Stage—CS4	112 (21.4)
CVM Stage—CS5	221 (42.3)
CVM Stage—CS6	77 (14.7)
Growth Phase—Early	71 (13.6)
Growth Phase—Peak	154 (29.4)
Growth Phase—Late	298 (57.0)

**Table 2:** External validation results on the external test set (*n* = 150), with 95% bootstrap confidence intervals (1000 resamples). (**A**) Overall phase-classifier performance; (**B**) per-class clinical metrics for the phase classifier; (**C**) end-to-end cascade performance for six-stage CVM classification.

**(A) Phase classifier performance**								
**Metric**			**Value**			**95% CI**		
Accuracy			0.9600			[0.9267, 0.9867]		
Macro F1			0.9191			[0.8302, 0.9745]		
Cohen’s κ			0.9236			[0.8591, 0.9742]		
Weighted κ			0.9511			[0.9079, 0.9836]		
Macro ROC-AUC			0.9975			[0.9935, 0.9998]		
**(B) Per-class clinical metrics for the phase classifier**								
**Phase**	**TP**	**FN**	**FP**	**TN**	**Sensitivity**	**Specificity**	**PPV**	**NPV**
Early	10	0	4	136	1.0000	0.9714	0.7143	1.0000
Peak	43	4	2	101	0.9149	0.9806	0.9556	0.9619
Late	91	2	0	57	0.9785	1.0000	1.0000	0.9661
**(C) End-to-end cascade performance (six-stage CVM classification)**								
**Metric**			**Value**			**95% CI**		
Accuracy			0.7933			[0.7267, 0.8533]		
Macro F1			0.7348			[0.5823, 0.8402]		
Cohen’s κ			0.7104			[0.6091, 0.7879]		
Weighted κ			0.9104			[0.8610, 0.9406]		
Macro ROC-AUC			0.9231			[0.8664, 0.9712]		

**Table 4:** Error-severity breakdown for the end-to-end cascade on the external test set (*n* = 150).

Error Type	Count	Percentage
Correct	119	79.3%
Off-by-one (adjacent)	29	19.3%
Off by two or more	2	1.3%
Within ±1 stage	148	98.7%

## Data Availability

The raw data supporting the conclusions of this article will be made available by the authors on request..

## Supplementary Materials

The following supporting information can be downloaded at: https://www.mdpi.com/article/10.3390/diagnostics16182886/s1, Figure S1. Confidence calibration, Figure S2. Inter-rater confusion matrix, Figure S3. YOLO performance summary, Figure S4. Bounding-box area distribution, Figure S5. Confusion matrices for the three phase-specific binary classifiers: early phase (CS1 vs. CS2), peak phase (CS3 vs. CS4), and late phase (CS5 vs.CS6), Table S1. Detector 5-fold performance, Table S2. Phase classifier internal CV, Table S3. Early phase-specific classifier, Table S4. Peak phase-specific classifier, Table S5. Late phase-specific classifier, Table S6. Distribution of cephalometric radiographs in the institutional dataset, Table S7. Inter-rater agreement statistics, Table S8. Cases of annotator disagreement, Table S9. Cross-validation summaries for all three Phase-Specific, Table S10. Composition of the external test set, Table S11. Early Phase-specific (CS1 vs. CS2) performance on the external test set, Table S12. Peak Phase-Specific (CS3 vs. CS4) performance on the external test set, Table S13. Late Phase-Specific (CS5 vs. CS6) performance on the external test set, Table S14. Per-class clinical metrics for the end-to-end six-stage cascade, Table S15. Top-10 highest-confidence wrong predictions, Table S16. Top-10 lowest-confidence wrong predictions. File S1. CLAIM 2024 Checklist. File S2. STARD 2015 Checklist. File S3. STROBE Statement.
