# Supplementary material for: Diagnostic Accuracy of a Machine Learning Model for Cervical Vertebra-Based Skeletal Maturity Assessment in Pediatric and Adolescent Patients Using Cephalometric Radiographs
Source: Diagnostics (Basel). 2026 Sep 8;16(18):2886. doi: 10.3390/diagnostics16182886 (PMC13605216; doi:10.3390/diagnostics16182886)
Supplement: Supplementary file 1 [file diagnostics-16-02886-s001.zip › File S1. CLAIM_2024_Checklist_.pdf]

Checklist for Artificial Intelligence in Medical Imaging (CLAIM): 2024 Update

Section/Topic	No.	Item	Page No
TITLE/ABSTRACT			
	1	Identification as a study of AI methodology, specifying the category of technology used (eg, deep learning)	1
ABSTRACT			
	2	Summary of study design, methods, results, and conclusions	1
INTRODUCTION			
	3	Scientific and/or clinical background, including the intended use and role of the AI approach	1–3
	4	Study aims, objectives, and hypotheses	3
METHODS			
Study Design	5	Prospective or retrospective study	3, 18
	6	Study goal	3
Data	7	Data sources	3–4, 6
	8	Inclusion and exclusion criteria	4
	9	Data preprocessing	6–7
	10	Selection of data subsets	5–6, 8–9
	11	De-identification methods	3, 19
	12	How missing data were handled	4–5, 10
	13	Image acquisition protocol	4, 6
Reference Standard	14	Definition of method(s) used to obtain reference standard	4–6
	15	Rationale for choosing the reference standard	4, 18
	16	Source of reference standard annotations	4–6
	17	Annotation of test set	6
	18	Measures of inter- and intrarater variability of features described by the annotators	4–5, 10–11
Data Partitions	19	How data were assigned to partitions	5–6, 8–9
	20	Level at which partitions are disjoint	5–6, 9
Testing Data	21	Intended sample size	5
Model	22	Detailed description of model	7–9
	23	Software libraries, frameworks, and packages	8, 10
	24	Initialization of model parameters	7–9
Training	25	Details of training approach	8–9
	26	Method of selecting the final model	8–9
	27	Ensembling techniques	7–9
Evaluation	28	Metrics of model performance	9–10
	29	Statistical measures of significance and uncertainty	9–15
	30	Robustness or sensitivity analysis	5–6, 8–10, 14–16
	31	Methods for explainability or interpretability	15–16 (descriptive analysis only)
	32	Evaluation on internal data	8–11
	33	Testing on external data	5–6, 9, 11–16
	34	Clinical trial registration	Not applicable (not a clinical trial/not registered)
RESULTS			
Data	35	Numbers of patients or examinations included and excluded	5, 10–11
	36	Demographic and clinical characteristics of cases in each partition	4, 6, 11–12
Model Performance	37	Performance metrics and measures of statistical uncertainty	11–15
	38	Estimates of diagnostic performance and their precision	12–15
	39	Failure analysis of incorrectly classified cases	12–16
DISCUSSION			
	40	Study limitations	18
	41	Implications for practice, including intended use and/or clinical role	16–18
OTHER INFORMATION			
	42	Provide a reference to the full study protocol or to additional technical details	19 (technical details; no full protocol link)
	43	Statement about the availability of software, trained model, and/or data	19 (data availability only)
	44	Sources of funding and other support; role of funders	19

Page numbers are indicated for each checklist item that is present in the manuscript.

Page numbers refer to the revised manuscript "Diagnostic Accuracy of a Machine Learning Model for Cervical Vertebrae-Based Skeletal Maturity Assessment in Pediatric andAdolescent Patients Using Cephalometric Radiographs" (21 numbered manuscript pages; PDF page 22 contains editorial comment details).
