# Supplementary material for: Diagnostic Accuracy of a Machine Learning Model for Cervical Vertebra-Based Skeletal Maturity Assessment in Pediatric and Adolescent Patients Using Cephalometric Radiographs
Source: Diagnostics (Basel). 2026 Sep 8;16(18):2886. doi: 10.3390/diagnostics16182886 (PMC13605216; doi:10.3390/diagnostics16182886)
Supplement: Supplementary file 1 [file diagnostics-16-02886-s001.zip › File S2. STARD_2015_Checklist_.pdf]

STARD 2015 — Essential items for reporting diagnostic accuracy studies

Section & Topic	No	Item	Page No
**TITLE OR ABSTRACT**			
	**1**	Identification as a study of diagnostic accuracy using at least one measure of accuracy (such as sensitivity, specificity, predictive values, or AUC)	1
**ABSTRACT**			
	**2**	Structured summary of study design, methods, results, and conclusions (for specific guidance, see STARD for Abstracts)	1
**INTRODUCTION**			
	**3**	Scientific and clinical background, including the intended use and clinical role of the index test	1–3
	**4**	Study objectives and hypotheses	3
**METHODS**			
Study design	**5**	Whether data collection was planned before the index test and reference standard were performed (prospective study) or after (retrospective study)	3, 11, 18
Participants	**6**	Eligibility criteria	4
	**7**	On what basis potentially eligible participants were identified (such as symptoms, results from previous tests, inclusion in registry)	3–4, 6
	**8**	Where and when potentially eligible participants were identified (setting, location and dates)	3
	**9**	Whether participants formed a consecutive, random or convenience series	3, 5, 18
Test methods	**10a**	Index test, in sufficient detail to allow replication	6–10
	**10b**	Reference standard, in sufficient detail to allow replication	4–6
	**11**	Rationale for choosing the reference standard (if alternatives exist)	4, 18
	**12a**	Definition of and rationale for test positivity cut-offs or result categories of the index test, distinguishing pre-specified from exploratory	7–9, 17
	**12b**	Definition of and rationale for test positivity cut-offs or result categories of the reference standard, distinguishing pre-specified from exploratory	4, 6
	**13a**	Whether clinical information and reference standard results were available to the performers/readers of the index test	6, 9, 14
	**13b**	Whether clinical information and index test results were available to the assessors of the reference standard	4
Analysis	**14**	Methods for estimating or comparing measures of diagnostic accuracy	9–10
	**15**	How indeterminate index test or reference standard results were handled	4–5, 11–12
	**16**	How missing data on the index test and reference standard were handled	4–5, 8, 10–12
	**17**	Any analyses of variability in diagnostic accuracy, distinguishing pre-specified from exploratory	9–10, 12–16, 18
	**18**	Intended sample size and how it was determined	5
**RESULTS**			
Participants	**19**	Flow of participants, using a diagram	10–11 (reported narratively; no flow diagram)
	**20**	Baseline demographic and clinical characteristics of participants	4, 6, 11–12
	**21a**	Distribution of severity of disease in those with the target condition	4, 11–12
	**21b**	Distribution of alternative diagnoses in those without the target condition	Not applicable
	**22**	Time interval and any clinical interventions between index test and reference standard	4 (implicit; no interval reported)
Test results	**23**	Cross tabulation of the index test results (or their distribution) by the results of the reference standard	12–14

Section & Topic	No	Item	Page No
	**24**	Estimates of diagnostic accuracy and their precision (such as 95% confidence intervals)	12–14
	**25**	Any adverse events from performing the index test or the reference standard	Not applicable
**DISCUSSION**			
	**26**	Study limitations, including sources of potential bias, statistical uncertainty, and generalisability	18
	**27**	Implications for practice, including the intended use and clinical role of the index test	16–18
**OTHER INFORMATION**			
	**28**	Registration number and name of registry	Not applicable (not registered)
	**29**	Where the full study protocol can be accessed	Not reported
	**30**	Sources of funding and other support; role of funders	19

Page numbers refer to the revised manuscript "Diagnostic Accuracy of a Machine Learning Model for Cervical Vertebrae-Based Skeletal Maturity Assessment in Pediatric andAdolescent Patients Using Cephalometric Radiographs" (21 numbered manuscript pages; PDF page 22 contains editorial comment details).
