# Supplementary material for: Diagnostic Accuracy of a Machine Learning Model for Cervical Vertebra-Based Skeletal Maturity Assessment in Pediatric and Adolescent Patients Using Cephalometric Radiographs
Source: Diagnostics (Basel). 2026 Sep 8;16(18):2886. doi: 10.3390/diagnostics16182886 (PMC13605216; doi:10.3390/diagnostics16182886)
Supplement: Supplementary file 1 [file diagnostics-16-02886-s001.zip › Supplementary Table S1.Detector 5-fold performance.pdf]

YOLOv8-nano cervical region detector performance under five-fold cross-validation on the institutional dataset (n. train ≈ 416, n. val ≈ 104 per fold).

Fold	mAP@0.5	mAP@0.5:0.95	Precision	Recall
1	0.9950	0.8116	0.9959	1.0000
2	0.9950	0.8433	0.9994	1.0000
3	0.9897	0.8218	0.9897	0.9904
4	0.9950	0.7487	0.9977	1.0000
5	0.9950	0.8286	0.9989	1.0000
**Mean ± SD**	**0.9939 ± 0.0024**	**0.8108 ± 0.0366**	**0.9963 ± 0.0039**	**0.9981 ± 0.0043**
