# Supplementary material for: Diagnostic Accuracy of a Machine Learning Model for Cervical Vertebra-Based Skeletal Maturity Assessment in Pediatric and Adolescent Patients Using Cephalometric Radiographs
Source: Diagnostics (Basel). 2026 Sep 8;16(18):2886. doi: 10.3390/diagnostics16182886 (PMC13605216; doi:10.3390/diagnostics16182886)
Supplement: Supplementary file 1 [file diagnostics-16-02886-s001.zip › Supplementary Table S10.Composition of the external test set.pdf]

Composition of the external test set (n = 150).

**Phase**	**Phase-Spicific**	**Images (n)**	**Phase Total**
Early	CS1	5	2*10
Early	CS2	5	
Peak	CS3	7	2*47
Peak	CS4	40	
Late	CS5	69	2*93
Late	CS6	24	
**Total**		**150**	**150**
