# Supplementary material for: Diagnostic Accuracy of a Machine Learning Model for Cervical Vertebra-Based Skeletal Maturity Assessment in Pediatric and Adolescent Patients Using Cephalometric Radiographs
Source: Diagnostics (Basel). 2026 Sep 8;16(18):2886. doi: 10.3390/diagnostics16182886 (PMC13605216; doi:10.3390/diagnostics16182886)
Supplement: Supplementary file 1 [file diagnostics-16-02886-s001.zip › Supplementary Table S11.Early Phase-specific (CS1 vs. CS2) performance on the external test set.pdf]

Early Phase-specific (CS1 vs. CS2) performance on the external test set (n = 10).

Metric	Value	95% CI
Accuracy	0.8000	[0.5000, 1.0000]
Macro F1	0.7917	[0.4444, 1.0000]
Cohen's κ	0.6000	[0.0909, 1.0000]
ROC-AUC	1.0000	[1.0000, 1.0000]
