# Supplementary material for: Diagnostic Accuracy of a Machine Learning Model for Cervical Vertebra-Based Skeletal Maturity Assessment in Pediatric and Adolescent Patients Using Cephalometric Radiographs
Source: Diagnostics (Basel). 2026 Sep 8;16(18):2886. doi: 10.3390/diagnostics16182886 (PMC13605216; doi:10.3390/diagnostics16182886)
Supplement: Supplementary file 1 [file diagnostics-16-02886-s001.zip › Supplementary Table S12.Peak Phase-Specific (CS3 vs. CS4) performance on the external test set.pdf]

Peak Phase-Specific (CS3 vs. CS4) performance on the external test set (n = 47).

**Metric**	**Value**	**95% CI**
Accuracy	0.9787	[0.9362, 1.0000]
Macro F1	0.9603	[0.8626, 1.0000]
Cohen’s κ	0.9207	[0.7283, 1.0000]
ROC-AUC	1.0000	[1.0000, 1.0000]
