# Supplementary material for: Diagnostic Accuracy of a Machine Learning Model for Cervical Vertebra-Based Skeletal Maturity Assessment in Pediatric and Adolescent Patients Using Cephalometric Radiographs
Source: Diagnostics (Basel). 2026 Sep 8;16(18):2886. doi: 10.3390/diagnostics16182886 (PMC13605216; doi:10.3390/diagnostics16182886)
Supplement: Supplementary file 1 [file diagnostics-16-02886-s001.zip › Supplementary Table S13.Late Phase-Specific (CS5 vs. CS6) performance on the external test set.pdf]

Late phase-specific (CS5 vs. CS6) performance on the external test set (n = 93).

**Metric**	**Value**	**95% CI**
Accuracy	0.7634	[0.6774, 0.8495]
Macro F1	0.7244	[0.6246, 0.8220]
Cohen's κ	0.4561	[0.2573, 0.6467]
ROC-AUC	0.8062	[0.6747, 0.9315]
