# Supplementary material for: Diagnostic Accuracy of a Machine Learning Model for Cervical Vertebra-Based Skeletal Maturity Assessment in Pediatric and Adolescent Patients Using Cephalometric Radiographs
Source: Diagnostics (Basel). 2026 Sep 8;16(18):2886. doi: 10.3390/diagnostics16182886 (PMC13605216; doi:10.3390/diagnostics16182886)
Supplement: Supplementary file 1 [file diagnostics-16-02886-s001.zip › Supplementary Table S14.Per-class clinical metrics for the end-to-end six-stage cascade.pdf]

Per-class clinical metrics for the end-to-end 6-stage cascade on the external test set (n = 150).

Stage	TP	FN	FP	TN	Sensitivity	Specificity	PPV	NPV
CS1	3	2	2	143	0.6000	0.9862	0.6000	0.9862
CS2	5	0	3	142	1.0000	0.9793	0.6250	1.0000
CS3	4	3	1	142	0.5714	0.9930	0.8000	0.9793
CS4	39	1	2	108	0.9750	0.9818	0.9512	0.9908
CS5	50	19	6	75	0.7246	0.9259	0.8929	0.7979
CS6	18	6	17	109	0.7500	0.8651	0.5143	0.9478
