# Supplementary material for: Diagnostic Accuracy of a Machine Learning Model for Cervical Vertebra-Based Skeletal Maturity Assessment in Pediatric and Adolescent Patients Using Cephalometric Radiographs
Source: Diagnostics (Basel). 2026 Sep 8;16(18):2886. doi: 10.3390/diagnostics16182886 (PMC13605216; doi:10.3390/diagnostics16182886)
Supplement: Supplementary file 1 [file diagnostics-16-02886-s001.zip › Supplementary Table S15.Top-10 highest-confidence wrong predictions.pdf]

Top-10 highest-confidence wrong predictions (overconfident errors).

True	Predicted	Phase conf	Sub conf	Overall conf
CS5	CS6	0.9283	0.7590	0.7046
CS5	CS6	0.9008	0.7559	0.6864
CS5	CS6	0.9096	0.7236	0.6582
CS5	CS6	0.8016	0.7488	0.6003
CS5	CS6	0.8306	0.7209	0.5988
CS5	CS6	0.9066	0.6498	0.5891
CS5	CS6	0.8399	0.6971	0.5856
CS5	CS6	0.8836	0.6379	0.5638
CS6	CS5	0.7613	0.7419	0.5648
CS5	CS6	0.7938	0.6913	0.5487
