# Supplementary material for: Diagnostic Accuracy of a Machine Learning Model for Cervical Vertebra-Based Skeletal Maturity Assessment in Pediatric and Adolescent Patients Using Cephalometric Radiographs
Source: Diagnostics (Basel). 2026 Sep 8;16(18):2886. doi: 10.3390/diagnostics16182886 (PMC13605216; doi:10.3390/diagnostics16182886)
Supplement: Supplementary file 1 [file diagnostics-16-02886-s001.zip › Supplementary Table S16.Top-10 lowest-confidence wrong predictions.pdf]

Top-10 lowest-confidence wrong predictions (uncertainty appropriately captured).

True	Predicted	Phase conf	Sub conf	Overall conf
CS4	CS3	0.4377	0.5896	0.2581
CS3	CS4	0.5275	0.5301	0.2797
CS4	CS5	0.5645	0.5138	0.2900
CS3	CS1	0.5960	0.5014	0.2989
CS6	CS5	0.4209	0.7469	0.3143
CS6	CS5	0.5225	0.6497	0.3394
CS3	CS1	0.6148	0.5550	0.3412
CS5	CS4	0.5377	0.6579	0.3539
CS5	CS4	0.7155	0.5465	0.3911
CS6	CS5	0.7155	0.5469	0.3911
