# Supplementary material for: Diagnostic Accuracy of a Machine Learning Model for Cervical Vertebra-Based Skeletal Maturity Assessment in Pediatric and Adolescent Patients Using Cephalometric Radiographs
Source: Diagnostics (Basel). 2026 Sep 8;16(18):2886. doi: 10.3390/diagnostics16182886 (PMC13605216; doi:10.3390/diagnostics16182886)
Supplement: Supplementary file 1 [file diagnostics-16-02886-s001.zip › Supplementary Table S2.Phase classifier internal CV.pdf]

Phase classifier per-fold performance with test-time augmentation.

Fold	Accuracy	Macro F1	Cohen's κ	Weighted κ	ROC-AUC
1	0.7885	0.7180	0.6239	0.7716	0.8894
2	0.7885	0.7096	0.6294	0.7786	0.9080
3	0.7961	0.7304	0.6395	0.7668	0.9071
4	0.7573	0.7070	0.5949	0.7788	0.9115
5	0.7379	0.6376	0.5540	0.7149	0.8935
**Mean ± SD**	**0.7736 ± 0.0250**	**0.7005 ± 0.0363**	**0.6083 ± 0.0346**	**0.7622 ± 0.0269**	**0.9019 ± 0.0098**
