# Supplementary material for: Diagnostic Accuracy of a Machine Learning Model for Cervical Vertebra-Based Skeletal Maturity Assessment in Pediatric and Adolescent Patients Using Cephalometric Radiographs
Source: Diagnostics (Basel). 2026 Sep 8;16(18):2886. doi: 10.3390/diagnostics16182886 (PMC13605216; doi:10.3390/diagnostics16182886)
Supplement: Supplementary file 1 [file diagnostics-16-02886-s001.zip › Supplementary Table S3.Early phase-specific classifier.pdf]

Early phase-specific classifier (CS1 vs. CS2) per-fold cross-validation performance (n = 71).

Fold	Accuracy	Macro F1	Cohen's κ	ROC-AUC
1	0.5714	0.5714	0.1429	0.6939
2	0.5714	0.5714	0.1429	0.6735
3	0.5714	0.5625	0.1429	0.5918
4	0.5714	0.5714	0.1429	0.5510
5	1.0000	1.0000	1.0000	1.0000
**Mean ± SD**	**0.6571 ± 0.1917**	**0.6554 ± 0.1927**	**0.3143 ± 0.3833**	**0.7020 ± 0.1765**
