# Supplementary material for: Diagnostic Accuracy of a Machine Learning Model for Cervical Vertebra-Based Skeletal Maturity Assessment in Pediatric and Adolescent Patients Using Cephalometric Radiographs
Source: Diagnostics (Basel). 2026 Sep 8;16(18):2886. doi: 10.3390/diagnostics16182886 (PMC13605216; doi:10.3390/diagnostics16182886)
Supplement: Supplementary file 1 [file diagnostics-16-02886-s001.zip › Supplementary Table S4.Peak phase-specific classifier.pdf]

Peak phase-specific classifier (CS3 vs. CS4) per-fold cross-validation performance (n = 154).

| Fold | Accuracy | Macro F1 | Cohen's κ | ROC-AUC |
|---|---|---|---|---|
| 1 | 0.8387 | 0.7237 | 0.4710 | 0.7880 |
| 2 | 0.6774 | 0.6310 | 0.2757 | 0.7446 |
| 3 | 0.6129 | 0.5571 | 0.1185 | 0.4293 |
| 4 | 0.7000 | 0.6000 | 0.2012 | 0.6023 |
| 5 | 0.6000 | 0.5500 | 0.1176 | 0.5227 |
| **Mean ± SD** | **0.6858 ± 0.0953** | **0.6124 ± 0.0704** | **0.2368 ± 0.1465** | **0.6174 ± 0.1499** |
