# Supplementary material for: Diagnostic Accuracy of a Machine Learning Model for Cervical Vertebra-Based Skeletal Maturity Assessment in Pediatric and Adolescent Patients Using Cephalometric Radiographs
Source: Diagnostics (Basel). 2026 Sep 8;16(18):2886. doi: 10.3390/diagnostics16182886 (PMC13605216; doi:10.3390/diagnostics16182886)
Supplement: Supplementary file 1 [file diagnostics-16-02886-s001.zip › Supplementary Table S5.Late phase-specific classifier.pdf]

Late phase-specific classifier (CS5 vs. CS6) per-fold cross-validation performance (n = 298).

Fold	Accuracy	Macro F1	Cohen's κ	ROC-AUC
1	0.7167	0.6135	0.2273	0.7156
2	0.7500	0.7151	0.4444	0.8341
3	0.6833	0.6480	0.3133	0.7386
4	0.6441	0.5583	0.1194	0.6455
5	0.6271	0.6182	0.3168	0.7061
**Mean ± SD**	**0.6842 ± 0.0506**	**0.6306 ± 0.0573**	**0.2842 ± 0.1204**	**0.7280 ± 0.0686**
