# Supplementary material for: Diagnostic Accuracy of a Machine Learning Model for Cervical Vertebra-Based Skeletal Maturity Assessment in Pediatric and Adolescent Patients Using Cephalometric Radiographs
Source: Diagnostics (Basel). 2026 Sep 8;16(18):2886. doi: 10.3390/diagnostics16182886 (PMC13605216; doi:10.3390/diagnostics16182886)
Supplement: Supplementary file 1 [file diagnostics-16-02886-s001.zip › Supplementary Table S6.Distribution of cephalometric radiographs in the institutional dataset..pdf]

Distribution of cephalometric radiographs in the institutional dataset.

**Phase**	**Six-stage CVM Classification**	**Images**	**Phase Total**
Early	CS1	37	2*71
Early	CS2	34	
Peak	CS3	42	2*154
Peak	CS4	112	
Late	CS5	221	2*298
Late	CS6	77	
**Total**		**523**	**523**
