# Supplementary material for: Diagnostic Accuracy of a Machine Learning Model for Cervical Vertebra-Based Skeletal Maturity Assessment in Pediatric and Adolescent Patients Using Cephalometric Radiographs
Source: Diagnostics (Basel). 2026 Sep 8;16(18):2886. doi: 10.3390/diagnostics16182886 (PMC13605216; doi:10.3390/diagnostics16182886)
Supplement: Supplementary file 1 [file diagnostics-16-02886-s001.zip › Supplementary Table S7.Inter-rater agreement statistics.pdf]

Inter-rater agreement statistics

Statistic	Estimate	95% CI	p-value
**Simple % agreement**	0.989		
**Cohen's kappa**			
Unweighted	0.984		<0.001
Linear weighted	0.988		<0.001
Quadratic weighted	0.989		<0.001
**Gwet's AC1**	0.987	0.98-1.00	<0.001
**Krippendorff's alpha**			
Ordinal	0.994		
Nominal	0.985		
**ICC (two-way agreement, single)**	0.989	0.99-0.99	<0.001

Note: All statistics are interpreted as "almost perfect" agreement per Landis & Koch (1977), where $\kappa > 0.80$ indicates almost perfect agreement.
