# Supplementary material for: Diagnostic Accuracy of a Machine Learning Model for Cervical Vertebra-Based Skeletal Maturity Assessment in Pediatric and Adolescent Patients Using Cephalometric Radiographs
Source: Diagnostics (Basel). 2026 Sep 8;16(18):2886. doi: 10.3390/diagnostics16182886 (PMC13605216; doi:10.3390/diagnostics16182886)
Supplement: Supplementary file 1 [file diagnostics-16-02886-s001.zip › Supplementary Table S8.Cases of annotator disagreement.pdf]

Cases of annotator disagreement

Image ID	Annotator 1	Annotator 2	CVM Stage	Stage distance
CVM-3	CS4	CS3	CS4	-1
CVM-78	CS5	CS4	CS4	-1
CVM-299	CS2	CS3	CS2	1
CVM-332	CS5	CS1	CS1	-4
CVM-408	CS2	CS1	CS1	-1
CVM-461	CS1	CS2	CS2	1
