# Supplementary material for: Diagnostic Accuracy of a Machine Learning Model for Cervical Vertebra-Based Skeletal Maturity Assessment in Pediatric and Adolescent Patients Using Cephalometric Radiographs
Source: Diagnostics (Basel). 2026 Sep 8;16(18):2886. doi: 10.3390/diagnostics16182886 (PMC13605216; doi:10.3390/diagnostics16182886)
Supplement: Supplementary file 1 [file diagnostics-16-02886-s001.zip › Supplementary Table S9.Cross-validation summaries for all three Phase-Specific.pdf]

consolidates the cross-validation summaries for all three Phase-Specific and the Combined cross-validation summaries for all three sub-stage specialists.

**Phase-Spicific**	**n**	**Mean Acc**	**SD Acc**	**Mean F1**	**SD F1**	**Mean AUC**
Early (CS1 vs. CS2)	69	0.6571	0.1917	0.6554	0.1927	0.7020
Peak (CS3 vs. CS4)	153	0.6858	0.0953	0.6124	0.0704	0.6174
Late (CS5 vs. CS6)	298	0.6842	0.0506	0.6306	0.0573	0.7280
